# Co-delivery of Siape1 and Melatonin by ^125^I-loaded PSMA-targeted Nanoparticles for the Treatment of Prostate Cancer

**DOI:** 10.2174/1574892818666230419081414

**Published:** 2023-05-11

**Authors:** Ying Liu, Lin Hao, Yang Dong, Bing-Zheng Dong, Xin-Lei Wang, Xing Liu, Zheng-Xiang Hu, Gao-Chuan Fang, Guang-Yue Wang, Jia-Xin Qin, Zhen-Duo Shi, Kun Pang

**Affiliations:** 1 Department of Urology, Xuzhou Central Hospital, Xuzhou Clinical School of Xuzhou Medical University, Jiangsu, China;; 2 Department of Central Laboratory, Xuzhou Central Hospital, Xuzhou Clinical School of Xuzhou Medical University, Xuzhou, Jiangsu, China;; 3 School of Life Sciences, Jiangsu Normal University, Jiangsu, China;; 4 Department of Graduate School, University of Jinzhou Medical University, Jinzhou, China;; 5 Department of Graduate School, University of Bengbu Medical College, Bengbu, China

**Keywords:** Apurinic/apyrimidinic endonuclease 1, polyarginine peptide, small interfering RNA, prostate-specific membrane antigen, pharmacokinetics, nanoparticles

## Abstract

**Background:**

Both apurinic/apyrimidinic endodeoxyribonuclease 1 (APE1) inhibition and melatonin suppress prostate cancer (PCa) growth.

**Objective:**

This study evaluated the therapeutic efficiency of self-assembled and prostate-specific membrane antigen (PSMA)-targeted nanocarrier loading ^125^I radioactive particles and encapsulating siRNA targeting APE1 (siAPE1) and melatonin for PCa.

**Methods:**

The linear polyarginine R12 polypeptide was prepared using Fmoc-Arg-Pbf-OH. The PSMA-targeted polymer was synthesized by conjugating azide-modified R12 peptide to PSMA monoclonal antibody (mAb). Before experiments, the PSMA-R12 nanocarrier was installed with melatonin and siAPE1, which were subsequently labeled by ^125^I radioactive particles. *In vitro* biocompatibility and cytotoxicity of nanocomposites were examined in LNCaP cells and *in vivo* biodistribution and pharmacokinetics were determined using PCa tumor-bearing mice.

**Results:**

PSMA-R12 nanocarrier was ~120 nm in size and was increased to ~150 nm by melatonin encapsulation. PSMA-R12 nanoparticles had efficient loading capacities of siAPE1, melatonin, and ^125^I particles. The co-delivery of melatonin and siAPE1 by PSMA-R12-^125^I showed synergistic effects on suppressing LNCaP cell proliferation and Bcl-2 expression and promoting cell apoptosis and caspase-3 expression. Pharmacokinetics analysis showed that Mel@PSMA-R12-^125^I particles had high uptake activity in the liver, spleen, kidney, intestine, and tumor, and were accumulated in the tumor sites within the first 8 h p.i., but was rapidly cleared from all the tested organs at 24 h p.i. Administration of nanoparticles to PCa tumors *in vivo* showed that Mel@PSMA-R12-^125^I/siAPE1 had high efficiency in suppressing PCa tumor growth.

**Conclusion:**

The PSMA-targeted nanocarrier encapsulating siAPE1 and melatonin is a promising therapeutic strategy for PCa and can provide a theoretical basis for patent applications.

## 1 INTRODUCTION

Prostate cancer (PCa) ranks 3^rd^ incidence of cancers worldwide and the most frequently diagnosed cancer in men [1]. The incidence and mortality of PCa are increasing, with 1.4 million new cases and 375,000 new deaths in 2021 worldwide [1-3]. The incidence of PCa is ethnic and genetic,and the highest incidence and mortality of PCa were reported in Northern and Western Europe, Australia/New Zealand [1-3]. The high incidence of PCa in Europe and the United States relies on locally promoted detection methods, such the prostate-specific antigen (PSA) testing [4-6]. The overdiagnosis of PSA-based screening reduced the rates of metastasis, recurrence, and death from PCa and improved treatment [6].

Prostate-specific membrane antigen (PSMA) is a type II integral membrane protein highly specific for prostate epithelial cells and PCa cells. PSMA is located in the cytoplasm of normal cells and converted into a membrane-bound protein in PCa cells [7]. The expression level of PSMA rises with tumor dedifferentiation and metastasis, and therefore it is a potential target for PCa-specific therapy. PSMA expression level is positively associated with preoperative PSA expression and early recurrence [8]. A shorter PSA doubling time predicts positive PSMA positron emission tomography (PET) results in patients with recurrent PCa [9]. Also, evidence shows that ^68^Ga-PSMA PET/computed tomography (CT) has a higher detection efficacy than ^18^F-fluoromethylcholine in PCa patients presenting biochemical or PSA failure [10, 11]. Based on the above evidence, several PSMA-targeting probes and nanoparticles have been established to achieve early diagnosis and treatment of PCa [12, 13]. Tumor-targeting nanoparticles are the effective co-delivery systems of drug molecules and small interfering RNA (siRNA) [14-17].

Melatonin is a hormone synthesized from tryptophan and is circadian-regulated, stimulated by darkness [18]. Melatonin is an inhibitor of tumor metabolism and a cytoprotective agent with low toxicity [19, 20]. It has an antiproliferative, antioxidant, and immunomodulatory effect [19, 20]. A major trend in patents from 2012 to 2014 is the use of melatonin or synthetic analogs in combination with other drugs to improve therapeutic efficacy or prevent pathological conditions [21, 22]. US8785501B2 (Anti-cancer tamoxifen-melatonin hybrid ligand; US Patent) claims that a melatonin-tamoxifen hybrid ligand comprises either tamoxifen or 4-hydroxytamoxifen in combination with melatonin unexpectedly improves the prevention and treatment of breast cancer [23]. CN1415309A (CN Patent) shows that the melatonin-selenium nanocomplex improves immune regulation and prevents cancer [24]. Also, CN114835759A (CN Patent) shows that the melatonin-platinum IV-carbon-nitrogen long-chain complex has the higher tumor-killing ability and cytotoxicity compared with cisplatin, especially the effect on sex hormone-related tumors [25]. At the same time, melatonin reduces cisplatin resistance in tumor cells in addition to the potential anti-tumor effect [25]. The production of melatonin decreases with age, which associates with an increased incidence of PCa [26]. Therefore, melatonin is a promising therapy for cancer treatment [27].

Apurinic/apyrimidinic endodeoxyribonuclease 1/redox factor-1 (APE1) is the main endonuclease of apurinic/apyrimidinic (AP), a DNA repair enzyme with AP activity. APE1 is a key enzyme in the base excision repair pathway, which is responsible for repairing small base lesions and the excision of AP sites, the most frequent pre-mutagenic lesions that can prevent normal DNA replication [28, 29]. APE1 is essential in activating oncogenic transcription factors, like nuclear factor-kappa B (NF-κB) and hypoxia-inducible factor (HIF)-1α. APE1 is highly expressed in a variety of tumors and is associated with poor prognosis, including PCa [30, 31]. The inhibition of APE1 induces apoptosis, pyroptosis, and necroptosis by promoting unrepaired DNA damage in tumor cells [28-31]; therefore, APE1 is a potential targeted therapy in the treatment of several human cancers. The delivery of drug molecules and siRNA targeting APE1 (siAPE1) by PSMA-targeted nanoparticles might be an effective treatment for PCa.

This study aimed to establish off-patent PSMA-targeted therapeutic nanoparticles that mediate siAPE1 and melatonin to achieve PCa therapy. The PSMA-targeted nanocarrier was prepared by conjugating polyarginine R12 polypeptide (azide-modified) to PSMA monoclonal antibody (mAb; PSMA-R12), which was then labeled by ^125^I radioactive particles and encapsulated melatonin and siAPE1. *In vitro* experiments were performed in LNCaP cells to detect the biocompatibility and cytotoxicity of self-assembled nanocomposites, and *in vivo* experiments were performed to determine the biodistribution and pharmacokinetics of them in PCa tumor-bearing mice. This study evaluated the efficiency of the PSMA-targeted systemic delivery of melatonin and siAPE1 for PCa treatment.

## 2 MATERIALS AND METHODS

### 2.1 Materials

Human PCa cell line LNCaP (luciferase-expressing or not) was obtained from American Type Culture Collection (ATCC, Manassas, VA, USA). BALB/c mice were purchased from Wuhan Institute of Biological Products Co., Ltd. (Wuhan, China). Amino resin, Fmoc-Arg-pbf-OH, piperidine, dimethylformamide (DMF), trifluoroacetic acid (TFA), dichloromethane (DCM), N,N-diisopropylethylamine (DIPEA), O-(1H-benzotriazol-1-yl)-N, N, N’, N’-tetramethyluronium hexafluorophosphate (HBTU), methanol, sodium dodecyl sulfate (SDS)-polyacrylamide gel, dimethyl sulfoxide (DMSO), acetonitrile, tris(3-hydroxypropyltriazolylmethyl)amine (THPTA), acrylic acid, copper sulfate (CuSO_4_), sodium ascorbate, and formaldehyde were purchased from Nanjing Shengxing Biotechnology Co., LTD (Nanjing, China). LysoTracker Green DND-26, siRNA against APE1 (siAPE1), negative control (NC) siRNA, and Cy3-labeled siAPE1 probe were purchased from Invitrogen (Carlsbad, CA, USA). Propiolic acid, RPMI 1640 medium, TRIzol^®^ reagent, 4-6-Diamidino-2-phenylindole (DAPI), fetal bovine serum (FBS), antibiotics (penicillin/streptomycin), and phosphate-buffered saline (PBS, pH = 7.4) were bought from Gibco BRL (Gaithersburg, MD, USA). ^125^I radioactive particles (half-life = 59.6 days) and a brachytherapy instrument were obtained from Shanghai GMS Pharmaceutical Co., Ltd (Shanghai, China). Melatonin, azido-PEG2-amine (N3-PEG-NH2), and sulfo-N-hydroxysuccini mide (sulfo-NHS) were bought from Sigma-Aldrich Co., Ltd (St. Louis, MO, USA). Hematoxylin and eosin (H&E) staining and RIPA lysis buffer were purchased from Solarbio (Beijing, China). The BCA assay kit, Cell Counting Kit-8 (CCK8) assay kit, and Annexin V-FITC Apoptosis Detection kit were purchased from Pierce (Bonn, Germany), Sangon (Shanghai, China), and BD Biosciences (Carlsbad, CA, USA), respectively. The ChamQ Universal SYBR qPCR master mix and HiScript II Reverse Transcription kit were purchased from Vazyme Biotech Co., Ltd. (Nanjing, China). PSMA mAb was purchased from Abcam (Cambridge, UK). Enhanced chemiluminescence (ECL) system and polyvinylidene fluoride (PVDF) membranes were obtained from Millipore Corp., (Bedford, MA, USA). Cell culture dishes of 6-well and 96-well were purchased from Corning (Corning, NY, USA).

### 2.2 Synthesis of Polyarginine R12 Polypeptide Polymer

R12 polypeptide was prepared using Fmoc-Arg-Pbf-OH and the solid-phase peptide synthesis methods. Removal of Fmoc from the resin was performed using DMF-piperidine (4:1). Excess arginine was cleaned using DMF. Subsequently, Fmoc-Arg-Pbf-OH (3 ×), HBTU (3 ×), and DIPEA (6 ×) were added in DMF for 2 h to bind the first arginine to resin. The reaction was repeated 11 times to synthesize polypeptide R12. The conjugation of hydrophobic residue to the polypeptide was performed using DMF/DCM (1:1), HBTU (3 ×), and DIPEA (6 ×) for 2 h. The peptide-attached resin was washed three times using DMF, DCM, and methanol, respectively. Peptides were cleaved from the resin using 83% TFA and were evaporated using a rotary flash evaporator. Peptide polymers were precipitated with cold ether and vacuum dried. Conjugation of N3-PEG-NH2 (1.0 mmol) to R12 (1.2 mmol) was carried out by incubating in HBTU (1.0 mmol) and DIEA (4.0 mmol) for 2 h at room temperature. Azide-modified peptides were then separated and purified using high-performance liquid chromatography (HPLC), with a gradient system from 0 to 100% acetonitrile and water (0.1% TFA, *v/v*; elution time of 1 h, 10.0 ml/min).

### 2.3 Conjugation of R12 Polypeptide to PSMA mAb

The conjugation of PSMA mAb to azide-modified peptides was mediated using propiolic acid. Firstly, propiolic acid was coupled to PSMA mAb (PSMA mAb/peptide = 1/10 molar ratio) and was activated by EDC (0.4 mmol, 102 mg) and sulfo-NHS (0.2 mmol, 22 mg). The conjugation of propiolic acid-PSMA mAb to R12 polypeptide polymer was achieved using the covalent coupling reaction by adding sodium ascorbate (200 mg), CuSO_4_ (3.2 mg), and THPTA (50 mg) at 30°C with stirring, with molar ratios of 1/10/0, 1/10/1, 1/10/2, 1/10/5, and 1/10/10 for PSMA mAb/azide-modified R12/ propiolic acid. HPLC monitored the reaction. Then, purification of the polymer was performed using HPLC.

### 2.4 Self-assembly of Melatonin/siAPE1-loaded and ^125^I-labeled Nanoparticles

The self-assembly of siAPE1 with PSMA-R12 polymer was performed by incubating PSMA-R12 with siAPE1 (100 pmol) at constant molar ratios of 5/1, 10/1, and 20/1. The self-assembly was completed in PBS for half an hour at 37°C. The self-assembly of melatonin with PSMA-R12/siAPE1 nanocomposites (Mel@PSMA-R12/siAPE1) was conducted at the molar ratios of 0/1, 10/1, 20/1, 30/1, and 40/1. The PSMA-R12 polymer encapsulating siAPE1, melatonin, or nothing was radiolabeled with ^125^I using the chloramine-T method as previously described [32]. The artistic representation of the synthetic process for PSMA-R12 nanoparticles and Mel@PSMA-R12-^125^I/siAPE1 nanocomposites is shown in Fig. (**1**).

### 2.5 Characterization of Nanoparticles

Gel electrophoresis was used to determine the optimum molar ratio of Mel@PSMA-R12-^125^I/siAPE1. Gel images were analyzed using a Gel Imager System (Vilber Lourmat, Collegien, France). Transmission electron microscopy (TEM) was used to analyze the size and shape of nanoparticles (JEM-100CX II; Joel, Tokyo, Japan). Also, dynamic light scattering (DLS) analysis was used to evaluate the hydrodynamic sizes of nanoparticles (Zetasizer Nano ZS-ZEN3600; Malvern Instruments; Malvern Instrument, Inc., London, UK).

### 2.6 *In Vitro* Drug Release

The *in vitro* drug release profile of melatonin loaded in PSMA-R12-^125^I nanoparticles (30/1 molar ratio) was detected using the reversed-phase HPLC. Mel@PSMA-R12-^125^I nanoparticles (concentration 2 mg/ml) were added in PBS (pH 5.0 and 7.4) and stirred at 400 rpm, 37°C. At each time interval, 0.5 ml of supernatant was taken, and 1 ml of PBS was added. The cumulative release of melatonin in PBS was determined at 1, 3, 6, 12, 24, 48, and 72 h using reversed-phase HPLC at room temperature (0.1% TFA, 1.0 ml/min). Experiments were performed in triplicates.

### 2.7 Cellular Localization

The cellular localization of siAPE1 and Mel@PSMA-R12-^125^I nanoparticles was determined using a laser scanning confocal microscope (LSCM; Leica Microsystems CMS GmbH, Wetzlar, Germany). Briefly, LNCaP cells were seeded in 6-well dishes (1 × 10^5^ cells/well) and incubated at 37°C. When reaching ~ 60% confluency, LNCaP cells were incubated with a medium containing Cy3-labeled siAPE1 and Mel@PSMA-R12-^125^I (30/1 molar ratio) for 12 h at 37°C. The LysoTracker Green DND-26 was used to label acidic late endosomes and lysosomes for 5 min. The fluorescent localization of Cy3 and LysoTracker Green in LNCaP cells was analyzed using LSCM.

### 2.8 *In Vitro* Cytotoxicity

The cytotoxicity of ^125^I, melatonin, and nanoparticles to LNCaP and DU145 cells was evaluated using the CCK8 assay. The 2 cell lines were placed into 96-well dishes (6 × 10^3^ cells/ well) and incubated in RPMI 1640 medium supplementing with FBS (10%) and antibiotics (penicillin/streptomycin, 1%) for 12 h at 37°C. Then, cells were incubated with RPMI 1640 medium containing FBS, antibiotics, and nanoparticles at various concentrations for 48 h. Cell viability was measured using a CCK8 assay kit according to the manufacturer’s instructions. Relative cell viability was assessed using the formula as follows: cell viability (%) = 100 × [(OD_Exp_-OD_CK_)/OD_CK_], where OD_CK_ and OD_Exp_ represent the mean OD value at 450 nm (OD_450_) of the control (CK) and experimental groups, respectively.

### 2.9 *In Vitro* Cell Apoptosis

The *in vitro* cell apoptosis was measured using Annexin V/PI staining and flow cytometric analysis (Becton Dickinson, San Jose, CA). LNCaP and DU145 cells were seeded in 6-well dishes (1×10^5^ cells/well) and incubated for 12 h at 37°C. Subsequently, cells were treated with Mel@PSMA-R12, Mel@PSMA-R12-^125^I, PSMA-R12-^125^I/siAPE1, and Mel@PSMA-R12-^125^I/siAPE1 nanoparticles (nanoparticles: 1 mg/ml, melatonin: 5 μg/ml, ^125^I radioactivity: 100 μCi/ml; PSMA-R12/siAPE1 molar ratio: 30/1) for 48 h at 37°C. Then, cells were harvested and washed three times with PBS. Cells were then resuspended in Annexin V binding buffer and incubated with Annexin V-FITC (5 μl, 15 min) and PI (5 μl, 15 min in the dark). The apoptotic cells (Annexin V-positive) were analyzed using FACScan flow cytometry (Becton Dickinson).

### 2.10 Establishment of PCa Mouse Model

According to National guidelines GB 14922.1-2001 after approved by Ethics Committee of Xuzhou Medical University (Ethics Committee Approval Number: 202104A34) Fifty BALB/c nude mice (22-25 g, 4-6 weeks old) were maintained in standard conditions (55-60% relative humidity, room temperature (18-30°C), four mice per cage, and a natural light-dark cycle) with free access to food and water. The PCa mouse model was constructed by the subcutaneous inoculation of LNCaP cells (luciferase-expressing, 1 × 10^6^ cells) in the right flank region of mice. When the volume of the primary tumor reached ~50 mm^3^, mice were randomly divided into five groups and were treated with tail vein injection of 50 μl of PBS (control; n = 10), Mel@PSMA-R12, Mel@PSMA-R12-^125^I, PSMA-R12-^125^I/siAPE1, and Mel@PSMA-R12-^125^I/siAPE1 nanoparticles (30/1 molar ratio; n = 10 in each group). Nude mice in the experimental groups were treated with melatonin (1 mg/kg body weight)/^125^I radioactive particles (0.6 mCi). Thirty days post-treatment, mice are measured by body weight, serum AST, ALT, ALP and Creatinine are determined by ELISA, followed by blood white blood cell count (WBC) and red blood cell count (RBC) by hemocytometer, to determine the effect of Mel@Nano-^125^I/siAPE1 on basic blood parameters such as body weight, liver and kidney function, and blood count. Serum IgG was determined using the ELISA assay to determine the effect of Mel@Nano-125I/siAPE1 on the overall immune response of animals. After then, all mice were sacrificed by cervical dislocation. Tumors were extracted and weighted. All the tissues were prepared for the analysis of Western blot, H&E staining, and immunohistochemical analysis. Biodistribution studies were performed using the *in vivo* imaging system and micro-PET/CT imaging system.

### 2.11 Biodistribution and Pharmacokinetics Studies

The qualitative real-time fluorescence imaging was used to evaluate the *in vivo* distribution of nanoparticles at 10, 20, and 30 d post the injection of nanoparticles using a small animal optical imaging system (Bruker *In-Vivo* FX Pro; Bruker, Billerica, MA, USA). Mice were isoflurane-anesthetized when testing. A nanoScan PET/CT preclinical imager (Mediso, Budapest, Hungary) was used to determine the uptake of ^125^I radioactive nanoparticles (tail vein injection, 7.4 MBq, in PBS) in PCa tumor-bearing mice. The pharmacokinetics of ^125^I radioactive nanoparticles was determined at 0.5, 1, 2, 3, and 4 h post the injection of the tracer. The uptake of the ^125^I-loaded particles in tissues was detected (at 2 h, 4 h, 8 h, 24 h, and 72 h post-injection, p.i.), and the activity uptake was expressed in %ID/g tissue. Pharmacokinetics of nanoparticles in the blood was detected at 0.5, 1, 3, 6, 12, 24, 48, and 72 h p.i.

### 2.12 Histopathological Analysis

The histopathological change in tumor tissues was evaluated using H&E staining. Briefly, tumor tissues were fixed in paraformaldehyde (4%), embedded in paraffin, deparaffinized, and hydrated in a series of xylene-ethanol. The 5 μm-thick sections were stained with H&E.

### 2.13 Quantitative Real-time PCR (qRT-PCR) Analysis of Genes

Total RNA was extracted from tumor tissues and LNCaP cells using the TRIzol^®^ reagent. Reverse transcription to the first-strand cDNA was performed using the HiScript II Reverse Transcription kit. PCR analysis was performed for the APE1 gene using a ChamQ Universal SYBR qPCR master mix and a qTower3 RT-PCR cycler (Analytik Jena, Jena, Germany). The amplification of APE1 mRNA was performed under the following conditions: one cycle of 95°C for 30 s and 40 cycles of 95°C for 10 s, 60°C for 20 s, and 72°C for 1 min. The relative expression level of APE1 mRNA was analyzed using the 2^-△△ct^ methods with the normalization to the Ct value of the internal reference gene β-actin.

### 2.14 Western Blot Analysis

Tumor samples and cells were lysed with RIPA lysis buffer following the manufacturer’s instructions. The quantification of protein samples was determined using a BCA kit. Protein separation was performed using the 10% SDS-polyacrylamide gel electrophoresis (PAGE) at 80 V and 100 mA for 50 min. Subsequently, proteins were transferred onto PVDF membranes. After blocking (in 5% skimmed milk) and washing, PVDF membranes were incubated with specific primary antibodies against Bcl-2, APE1, Caspase 3, β-actin (1: 1500), and GAPDH (1: 1000) at 4°C overnight. Subsequent secondary incubation with horseradish peroxidase-conjugated secondary antibody (1: 5000) was performed at room temperature for 1 h. Protein expression was analyzed using the ECL and Image-Pro Plus 6.0 software (Media Cybernetics Inc., Bethesda, MD, USA).

### 2.15 Statistical Analysis

All cellular experiments were performed in triplicates and data were expressed as mean ± standard deviation. The differences between groups were analyzed using the unpaired *t*-test, and those across more than three groups were analyzed using the one-way Analysis of Variance (ANOVA) test (correction with the Holm-Sidak test). GraphPad Prism software (version 8; GraphPad Prism Software Inc., San Diego, USA) was used for the statistical analysis. The difference with a *p*-value of less than 0.05 was statistically significant.

## 3 RESULTS

### 3.1 Characterization and Release Behavior of Melatonin-loaded PSMA-R12 Nanoparticles

**Fig. (2):**
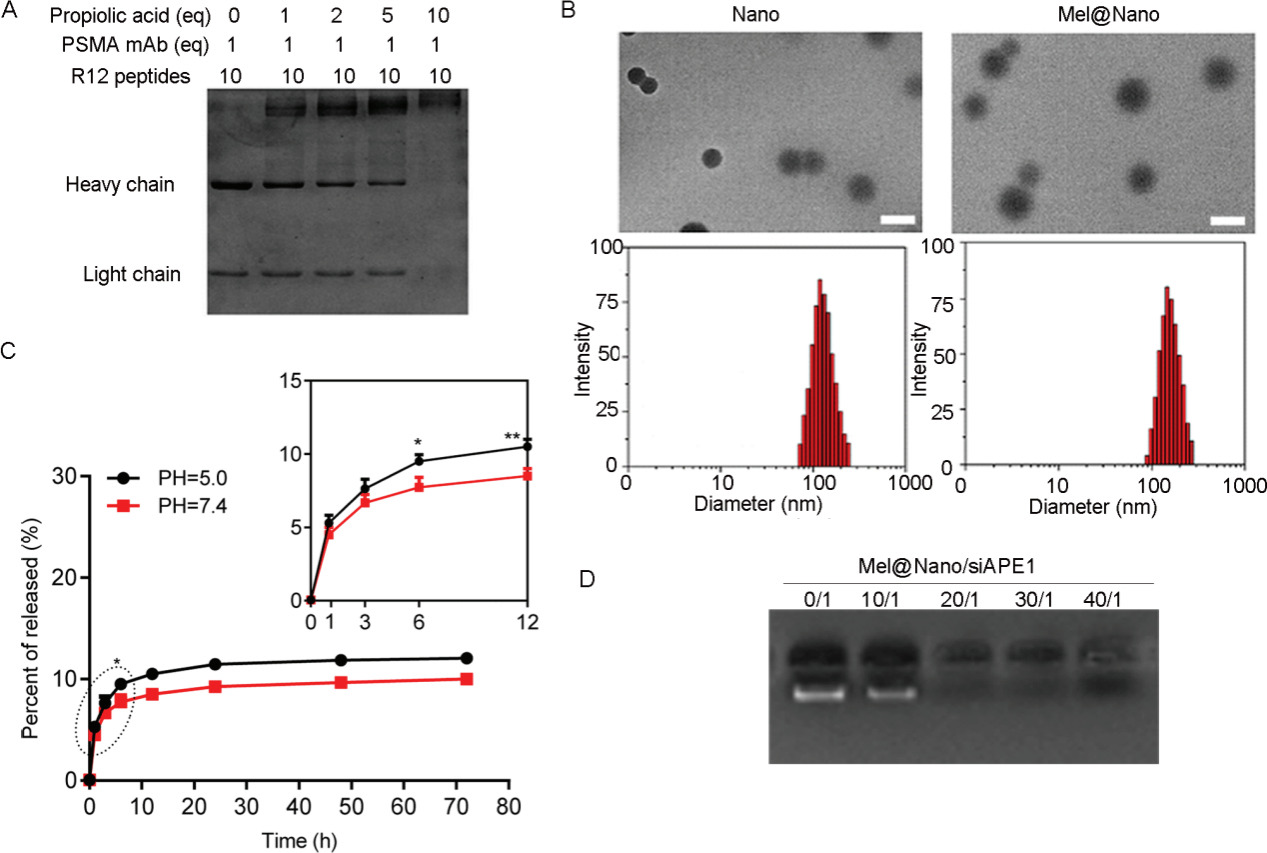
Characterization of self-assembled nanoparticles PSMA-R12. (**A**) gel electrophoresis showing the molar ratios of PSMA mAb/azide-modified R12/propiolic acid. (**B**) transmission electron microscopy (TEM) and dynamic light scattering (DLS) analysis of the PSMA-R12 nanoparticles with and without of loading of melatonin. Scale bar = 100 nm. (**C**) cumulative release profiles of melatonin-loaded PSMA-R12 nanoparticles in PBS (pH 5.0 and 7.4). This profile was obtained by reversed-phase high-performance liquid chromatography analysis. (**D**) gel electrophoresis showing the molar ratios of PSMA-R12 nanoparticles to free siAPE1. Data were expressed as mean ± standard deviation. Differences between groups were analyzed using the unpaired *t*-test. *p < 0.05 and **p < 0.01 with the comparison between pH 5.0 and 7.4 at tested time intervals.

**Fig. (3):**
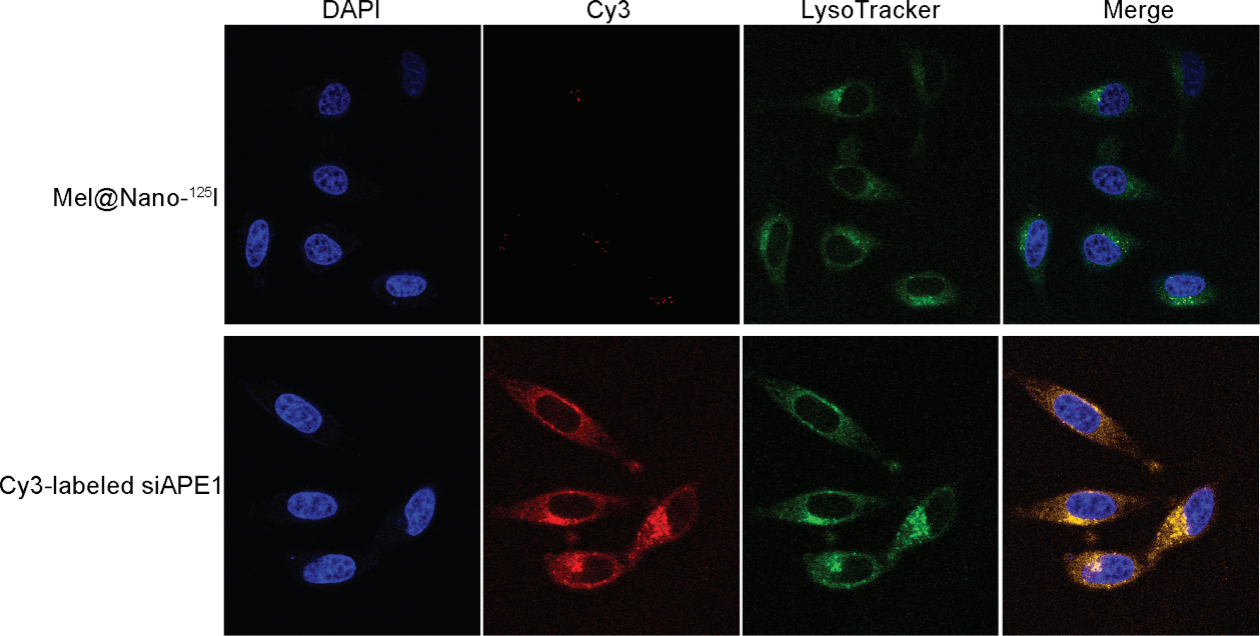
Fluorescence images showing the localization of nanoparticles in LNCaP cells. The cellular localization of Cy3-labeled siAPE1 and Mel@PSMA-R12-^125^I nanoparticles (1/30 molar ratio) was determined using a laser scanning confocal microscope. LysoTracker Green DND-26 was used to label acidic late endosomes and lysosomes, and nuclear was stained using DAPI.

The structure and construction principles of nanoparticles are shown in Fig. (**1**). Gel electrophoresis showed that the optimum molar ratio of PSMA mAb/azide-modified R12/propiolic acid was 1/10/10 (Fig. **2A**), at which the PSMA mAb was completely encapsulated by R12 peptides. The TEM analysis showed that the PSMA-R12 nanoparticles exhibited a uniform spherical structure and the size of PSMA-R12 nanoparticles was increased from ~120 nm to ~150 nm with the loading of melatonin (5 μg/ml; Fig. **2B**). As shown in Fig. (**2C**), melatonin-loaded PSMA-R12 nanoparticles showed a slow and sustained-release behavior that lasted at least 72 h in PBS. About 10.0% melatonin was released from nanoparticles at the end of 72 h (pH = 7.4). Reversed-phase HPLC analysis showed that melatonin was rapidly released at the first 6 h and a faster drug release rate was observed at pH = 5.0 than pH = 7.4 (9.5% *vs*. 7.7% at 6 h, *p* < 0.05; and 10.5% *vs*. 8.5% at 12 h, *p* < 0.01; Fig. **2C**). Gel electrophoresis showed that the self-assembled PSMA-R12 nanoparticles efficiently loaded free siAPE1. Free siAPE1 disappeared when the molar ratio of Mel@PSMA-R12 (Mel@Nano) to siAPE1 reached 20/1 (Fig. **2D**). We analyzed the localization of Cy3-labeled siAPE1 and ^125^I-labeled nanoparticles in LNCaP cells using LSCM, which tracked the fluorescence signals of Cy3-labeled siAPE1 and LysoTracker Green (DND-26)-labeled endosomes and lysosomes (Fig. **3**). Both siAPE1 and self-assembled nanoparticles localize on endosomes and lysosomes in the cytoplasm (Fig. **3**), showing PSMA-R12 could effectively combine with siAPE1.

### 3.2 Biocompatibility and Cytotoxicity of Melatonin, ^125^I, and PSMA-R12 Nanoparticles

**Fig. (4):**
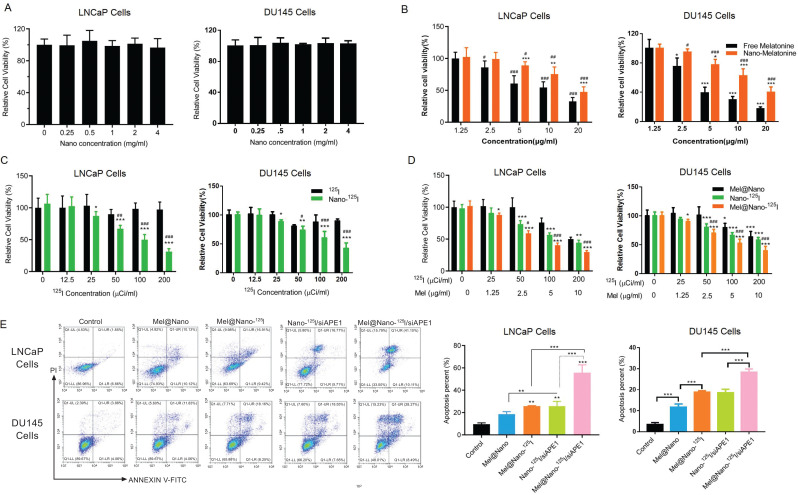
Study of *in vitro* cytotoxicity in LNCaP and DU145 cells. (**A**) biocompatibility of PSMA-R12 nanoparticles in LNCaP and DU145 cells. (**B**) and (**C**) cell viability of LNCaP and DU145 cells cultured with medium containing free melatonin and ^125^I radioactive particles or encapsulated with PSMA-R12 nanoparticles (Mel@Nano, Nano-^125^I, and Mel@Nano-^125^I) at tested concentrations. (**D**) melatonin/^125^I-loaded nanoparticles enhanced the cytotoxicity of melatonin and ^125^I to LNCaP and DU145 cells. Cells were cultured in medium and stimulus for 48 h, and cell viability was determined using the CCK8 assay. (**E**), the apoptosis of cells was assessed using the Annexin V/PI staining and flow cytometric analysis (nanoparticles: 1 mg/ml, melatonin: 5 μg/ml, ^125^I radioactivity: 100 μCi/ml; PSMA-R12/siAPE1 molar ratio: 30/1). Data were expressed as mean ± standard deviation. The differences between groups were analyzed using the unpaired *t*-test, and differences across more than three groups were analyzed using the one-way Analysis of Variance (ANOVA) test (correction with the Holm-Sidak test). *, **, and *** indicates *p* < 0.05, *p* < 0.01, and *p* < 0.001 compared with the corresponding control groups free melatonin in figure B, free ^125^I particles in Fig. (**C**), Mel@Nano in Fig. (**D**), and control in Fig. (**E**). ^#^, ^##^, and ^###^ represents *p* < 0.05, *p* < 0.01, and *p* < 0.001 compared with the corresponding control groups: 1.25 μg/ml in figure B, 0 μCi/ml ^125^I in Fig. (**C**), and Nano-^125^I groups at all tested concentrations in Fig. (**D**).

The biocompatibility of PSMA-R12 nanoparticles was assessed in both LNCaP and DU145 cells. CCK8 assay showed that LNCaP and DU145 cells' cell viability was not influenced by nanoparticles with concentrations ranging from 0.25 to 4 mg/ml (Fig. **4A**). The cytotoxicity of melatonin showed to both LNCaP and DU145 cells increased with concentrations ranging from 1.25 to 20 μg/ml. Notably, the encapsulation of melatonin in PSMA-R12 nanoparticles reduced melatonin-induced cytotoxicity to the cells (Fig. **4B**). Also, ^125^I-loaded nanoparticles showed higher cytotoxicity than free ^125^I radioactive particles (Fig. **4C**). Furthermore, the CCK8 analysis showed that ^125^I- and ^125^I/melatonin-loaded nanoparticles (Mel@Nano-^125^I), especially the latter, had significantly higher cytotoxicity to LNCaP and DU145 cells than melatonin-loaded nanoparticles (Mel@Nano; Fig. **4D**). These results showed that the co-delivery of melatonin and ^125^I radioactive particles by PSMA-R12 enhanced the therapeutic efficiencies of melatonin and ^125^I-loaded PSMA-R12 in CaP cells.

### 3.3 Co-delivery of siAPE1 and Melatonin by Nanoparticles Enhanced the Apoptosis of LNCaP and DU145 Cells

**Fig. (5):**
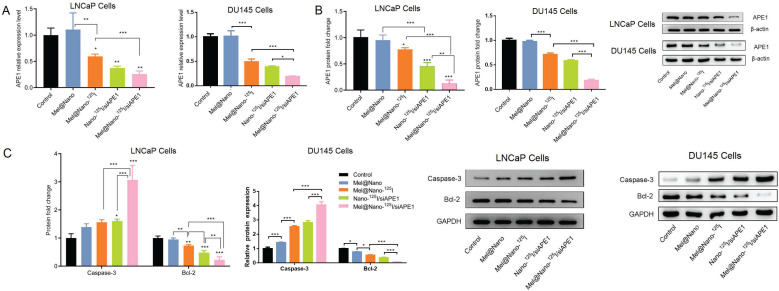
Expression of APE1, caspase 3, and Bcl-2 proteins and in LNCaP and DU145 cells. Cells were cultured in medium and stimulus (nanoparticles: 1 mg/ml, melatonin: 5 μg/ml, ^125^I radioactivity: 100 μCi/ml; PSMA-R12/siAPE1 molar ratio: 30/1) for 48 h, and the expression of APE1 mRNA (**A**) and protein (**B**) and the expression of caspase 3 and Bcl-2 proteins (**C**) were determined using the PCR analysis (**A**) and western blot analysis (**B** and **C**), respectively. Differences across more than three groups were analyzed using the one-way Analysis of Variance (ANOVA) test (correction with the Holm-Sidak test). *, ** and *** indicates *p* < 0.05, *p* < 0.01, and *p* < 0.001 when compared with the corresponding control groups.

We analyzed the therapeutic efficiencies of melatonin and ^125^I particles with and without siAPE1 co-delivery in LNCaP and DU145 cells using the Annexin V/PI staining analysis. Flow cytometry showed that the percent of apoptotic cells was increased in the order of Mel@Nano, Mel@Nano-^125^I, Nano-^125^I/siAPE1, and Mel@Nano-^125^I/siAPE1 (Fig. **4E**). As shown in Fig. (**4E**), the apoptotic cell percentage was increased by ^125^I-loaded PSMA-R12 encapsulating melatonin or siAPE1 when compared with the control (*p* < 0.01, Fig. **4E**). Also, the co-delivery of melatonin, siAPE1, and ^125^I particles by PSMA-R12 (Mel@Nano-^125^I/siAPE1) had the highest efficiency in promoting the apoptosis of LNCaP and DU145 cells when compared with PSMA-R12-^125^I encapsulating melatonin or siAPE1 (*p* < 0.01). The levels of siAPE1 mRNA and protein were decreased in the order of Mel@Nano-^125^I, Nano-^125^I/siAPE1, and Mel@Nano-^125^I/siAPE1 (Figs. **5A** and **B**), and so was Bcl-2 (Fig. **5C**). The expression pattern of the Caspase 3 protein in both LNCaP and DU145 cells was consistent with the percent of apoptotic cells, and Mel@Nano-^125^I/siAPE1 had the highest efficiency in promoting Caspase 3 expression than the others (Fig. **5C**).

### 3.4 Biodistribution and Pharmacokinetics of Self-assembled Nanocomposites in PCa Tumor-bearing Mice

**Fig. (6):**
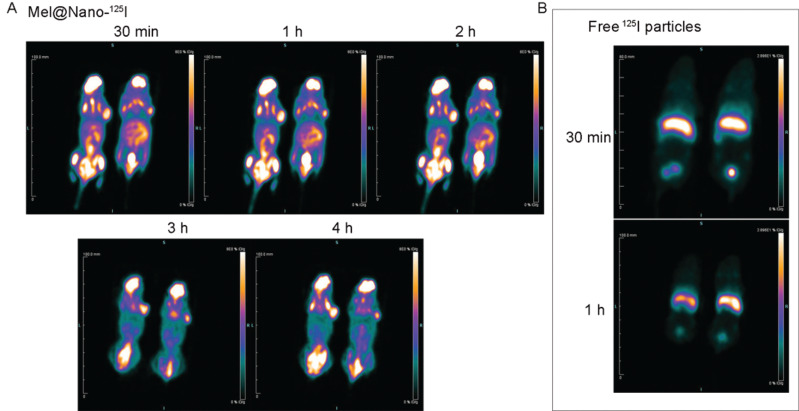
PET/CT preclinical imaging of nanocomposites *in vivo*. (**A**) *in vivo* time-dependent *in vivo* imaging of tumor-bearing mice at 0.5, 1, 2, 3, and 4 h post tail vein injection of PSMA-R12 nanoparticles encapsulated melatonin (Mel, 1 mg/kg body weight) and ^125^I radioactive particles (0.6 mCi). (**B**) *in vivo* imaging tumor-bearing mice at 0.5 and 1 h post tail vein injection of free ^125^I radioactive particles (0.6 mCi).

PET/CT scanning imaging showed that Mel@Nano-^125^I accumulated in the heart, liver, intestine, bladder, brain, kidney, and tumors (Fig. **6A**). However, free ^125^I radioactive particles only accumulated in the liver (Fig. **6B**). PET/CT imaging revealed the rapid clearance of the ^125^I tracer in the liver and kidney within the first 3 h p.i., showing the predominantly hepatic and renal excretion of the self-assembled nanocomposites (Fig. **6A**).

**Fig. (7):**
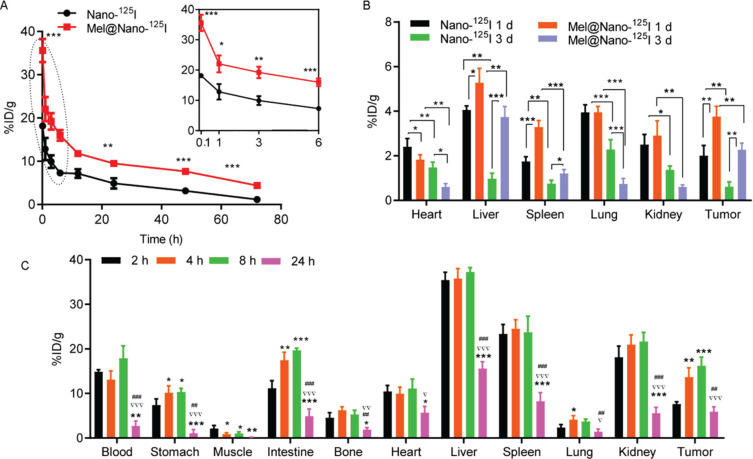
Pharmacokinetics of Mel@PSMA-R12/siAPE1 nanocomposites in tumor-bearing mice. (**A**) the pharmacokinetics of Mel@Nano-^125^I and Nano-^125^I in the blood of tumor-bearing mice. (**B**) the comparative ^125^I activity biodistribution in the heart, liver, spleen, lung, kidney, and tumor tissues after administration of nanocomposites in tumor-bearing mice. (**C**) the time-dependent biodistribution of Mel@Nano-^125^I activity in selected tissues in mice bearing prostatic tumors. Data were expressed as mean ± standard deviation. The differences between groups were analyzed using the unpaired *t*-test, and differences across more than three groups were analyzed using the one-way Analysis of Variance (ANOVA) test (correction with the Holm-Sidak test). *, ** and *** indicates *p* < 0.05, *p* < 0.01, and *p* < 0.001 when compared with the corresponding control groups (2 h group in Fig. **C**). ^##^ and ^###^ represents *p* < 0.05, *p* < 0.01, and *p* < 0.001 *versus* 4 h. ^∇^, ^∇∇^, and ^∇∇∇^ shows *p* < 0.05, *p* < 0.01, and *p* < 0.001 *versus* 8 h.

Pharmacokinetics analysis also showed that the ^125^I tracer was rapidly cleared from the blood pool within 72 h (Fig. **7A**). The encapsulation of melatonin reduced the clearance of Nano-^125^I particles, which showed high activity uptake in the liver (4.1 ± 0.2% ID/g), lung (3.9 ± 0.3% ID/g), kidney (2.5 ± 0.5% ID/g), and tumor (2.0 ± 0.5% ID/g, all data at 1 h p.i.; Fig. **7B**). Notably, we found that the encapsulation of melatonin promoted its uptake in the liver (from 4.1 ± 0.2% ID/g to 5.3 ± 0.6% ID/g, *p* < 0.05), spleen (from 1.75 ± 0.2% ID/g to 3.3 ± 0.3% ID/g, *p* < 0.001), and tumor (from 2.0 ± 0.5% ID/g to 3.8 ± 0.5% ID/g, *p* < 0.01; Fig. **7B**), but decreased its uptake in the heart (from 2.4 ± 0.4% ID/g to 1.8 ± 0.1% ID/g, all data at 1 h p.i., *p* < 0.05; Fig. **7B**). Nanoparticles were excreted partially from all organs at 3 d p.i. Furthermore, the pharmacokinetics analysis indicated that the Mel@Nano-^125^I particles showed predominantly uptake in the liver (37.2 ± 1.0% ID/g), spleen (23.7 ± 3.6% ID/g), kidney (21.6 ± 2.1% ID/g), intestine (19.6 ± 0.5% ID/g), and tumor (16.2 ± 1.9% ID/g) within the first 8 h p.i. (Fig. **7C**). Mel@PSMA-R12-^125^I gradually accumulated in the tumor sites, stomach, and intestine during the first 8 h p.i., but was rapidly cleared from all the tested organs except for the tumors at 24 h p.i.

### 3.5 Targeted Delivery and Therapeutic Function of Nanocomposites

**Fig. (8):**
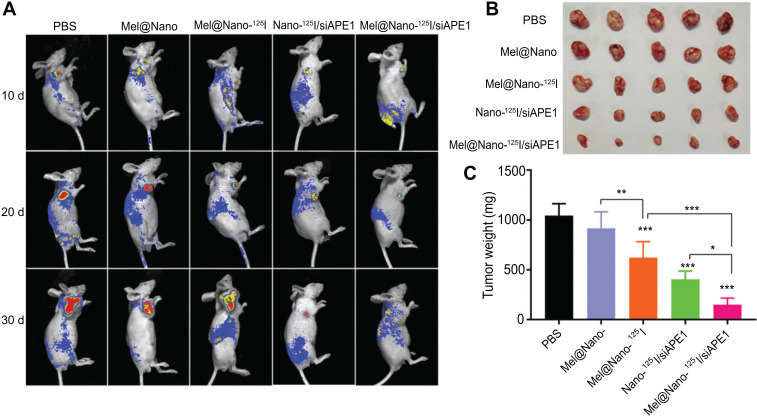
Targeted delivery, *in vivo* distribution and and biodistribution of nanocomposites in prostatic tumor-bearing mice. (**A**) the *in vivo* fluorescence imaging of nanoparticles tumor-bearing mice at 10, 20, and 30 d tail vein injection of PSMA-R12 nanoparticles. (**B**) images showing the influence of nanoparticles on *in vivo* tumor growth. (**C**) the differences in the weight of prostatic tumors obtained from tumor-bearing mice treated with nanoparticles for 30 d. Differences across more than three groups were analyzed using the one-way Analysis of Variance (ANOVA) test (correction with the Holm-Sidak test). *, ** and *** indicates *p* < 0.05, *p* < 0.01, and *p* < 0.001 compared with the corresponding control groups.

The *in vivo* time-dependent fluorescent images of nanoparticles are shown in Fig. (**8A**), that nanocomposites exhibit effective accumulation in the tumor sites (LNCaP-luc cells) at 30 d p.i. Nanoparticles suppressed PCa tumor growth *in vivo* (Figs. **8A**-**C**). The co-delivery of melatonin, ^125^I particles, and siAPE1 by PSMA-R12 nanoparticles enhanced the therapeutic efficiency of the single delivery of these particles. The weight of *in vivo* tumors in the Mel@Nano-^125^I/siAPE1 group (150.0 ± 65.1 g) was significantly lower than that in the Nano-^125^I/siAPE1 (403.4 ± 83.4 g, *p* < 0.05) and Mel@Nano-^125^I (622.8 ± 159.1 g, *p* < 0.001) groups (Fig. **8C**). These results showed that targeted co-delivery of melatonin and siAPE1 by ^125^I-loaded PSMA-R12 nanoparticles enhanced the therapeutic efficiency of the single delivery.

### 3.6 Cytotoxicity and Tissue Tolerance of Nanocomposites

**Fig. (9):**
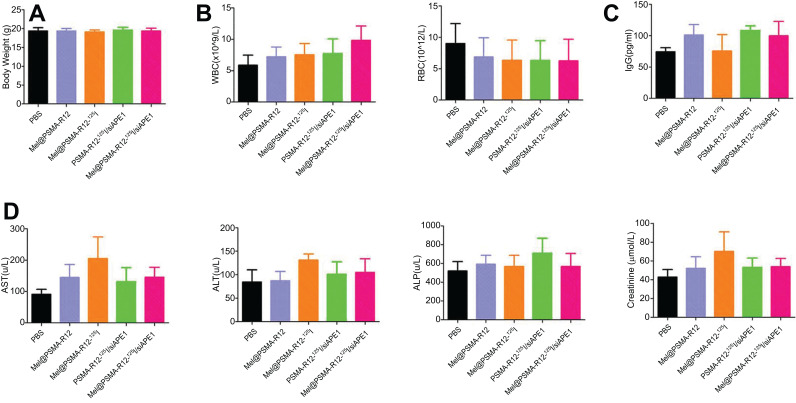
Effects on body weight, hematologic indicators, immune indicators, and liver and kidney function of nanocomposites in prostatic tumor-bearing mice. (**A**) comparison of body weight of tumor-bearing mice treated with nanoparticles and the control mice for 30 d. (**B**) the comparison of major blood indicators among groups. (**C**) comparison of serum IgG among the groups. (**D**) comparison of major parameters of liver and kidney function such as AST, ALT, ALP and Creatinine among the groups. Differences across more than three groups were analyzed using the one-way Analysis of Variance (ANOVA) test (correction with the Holm-Sidak test).

Before the end of the experiment (30 days), there was no significant difference in mouse body weight between the Mel@Nano-^125^I/siAPE1 group and the control groups (Fig. **9A**). At the same time, the WBC and RBC measured by the blood cell analyzer did not have significant differences among the groups (Fig. **9B**), suggesting that Mel@Nano-^125^I/siAPE1 had no significant effect on basic blood indicators in mice. The results of ELISA showed that there was no significant difference in serum IgG between groups, suggesting that Mel@Nano-^125^I/siAPE1 had no significant effect on the overall immunity of animals (Fig. **9C**). The liver and kidney functions measured by ELISA, such as AST, ALT, ALP and Creatinine, had no significant difference between the Mel@Nano-^125^I/siAPE1 group and the control groups (Fig. **9D**), suggesting that Mel@Nano-^125^I/siAPE1 had no significant effect on the liver and kidney function.

**Fig. (10):**
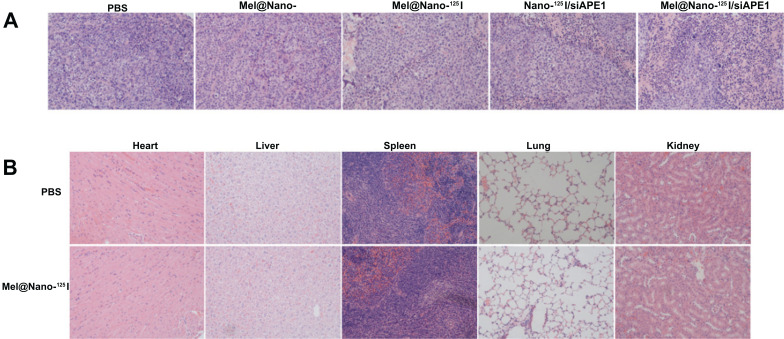
Histological changes in tumor and organs in prostatic tumor-bearing mice influenced by nanocomposites. (**A**) The hematoxylin and eosin (H&E) staining of tumor tissue sections after treatment. Magnification × 40. (**B**), The hematoxylin and eosin (H&E) staining of sections after treatment with PSMA-R12 nanoparticles for 30 d. Magnification × 40.

H&E staining showed that most of the tumor cells in the control groups were normal in shape without obvious cell apoptosis or necrosis, but tumors in the Mel@Nano-^125^I/siAPE1 group showed severe tumor cell apoptosis or necrosis, with the broken nucleus and diffused cytoplasm (Fig. **10A**). Moreover, no significant histological changes were found in the heart, liver, spleen, lung, and kidney (Fig. **10B**). These results showed that targeted co-delivery of melatonin and siAPE1 by ^125^I-loaded PSMA-R12 nanoparticles have no obviously normal cell and tissue toxicity.

## 4 DISCUSSION

RNAi is an effective regulation control for posttranscriptional gene expression, but the application of siRNA therapy is hampered because of the lack of targeting ability. Recently, tumor-targeting nanoparticles have been designed to improve the targeting ability and intracellular translocation efficiency of siRNA [33]. Great development has been made in nanoparticles to establish efficient delivery systems of siRNA against oncogenes and drug molecules for the treatment of cancers [34-37]. PSMA-targeted nanoparticles exhibited prominent anticancer activity in PCa cells [12, 13, 38]. We established the PSMA-targeted Mel@PSMA-R12-^125^I nanoparticles with the efficacy of encapsulating melatonin and APE1 siRNA. Nanoparticles were prepared by conjugating azide-modified R12 polypeptide to PSMA mAb, encapsulating melatonin, siAPE1, and ^125^I radioactive particles. Naked nanoparticles PSMA-R12 exhibited good biocompatibility and non-cytotoxicity in LNCaP and DU145 cells. Also, the encapsulations of melatonin, siAPE1, and ^125^I radioactive particles were efficient therapies for the treatment of PCa, as the Mel@PSMA-R12-^125^I/siAPE1 nanocomposites inhibited cell proliferation in LNCaP and DU145 cells *in vitro* and suppressed PCa tumor growth *in vivo*. These results suggested that Mel@PSMA-R12-^125^I/siAPE1 was a promising PCa-targeted therapy for PCa treatment.

Melatonin functions antiproliferative, antioxidant, and immunomodulating effects [19, 20]. Evidence-based studies showed that melatonin's antitumor and antiproliferative effects in PCa cells are medicated by increasing cAMP [39] and inhibiting NF-κB transcriptional activity and integrin α_2_β_1_ expression *via* the MT1 signaling pathway [40]. A clinical observation indicated daily melatonin consumption stabilized PSA levels [41]. Also, evidence shows that male workers who had night shift work experience at least one year are at high risk of PCa [42-44]. There are promising relationships between melatonin and the risk and prognosis of several human cancers [45]. A retrospective study of 955 PCa patients by Zharinov *et al*. [20] proved that melatonin administration improved median overall survival in patients with poor prognosis (153.5 *versus* 64.0 months). The multivariate analysis indicated that melatonin usage was an independent prognostic factor because it reduced the risk of death of PCa patients by more than twice. Our present study confirmed that melatonin showed antiproliferative and antitumor effects in PCa cells. Free or encapsulated melatonin by PSMA-R12 nanoparticles inhibited LNCaP and DU145 cell proliferation significantly. We also observed that the encapsulation of melatonin by PSMA-R12-^125^I decreased the cardiotoxicity in mice because Mel@PSMA-R12-^125^I particles had lower uptake activities in the heart compared with naked PSMA-R12-^125^I. The data showed that melatonin, naked or encapsulated by nanocarriers, was a potential treatment option for PCa.

APE1 functions as an oncogene whose expression is associated with poor prognosis in PCa [30, 31]. APE1 activates oncogenic transcription factors like NF-κB and HIF-1α. APE1 inhibition-mediated antitumor effect is induced by promoting unrepaired DNA damage, apoptosis, pyroptosis, and necroptosis in tumor cells [28-31]. Our study showed that the delivery of siAPE1 by PSMA-targeted therapeutic nanoparticles (Mel@PSMA-R12-^125^I) might be an effective treatment for PCa because it efficiently suppressed *in vitro* PCa cell proliferation and *in vivo* PCa tumor growth. Compared with the single delivery of siAPE1 or melatonin, their co-delivery by ^125^I-loaded PSMA-R12 nanoparticles had superior efficiencies in inhibiting Bcl-2 expression and promoting apoptosis in PCa cells and suppressing PCa tumor growth *in vivo*.

The biodistribution (PET/CT scanning) and pharmacokinetics analysis showed that self-assembled nanocomposites had good biodegradability and PCa tumor cell-targeting properties. Mel@PSMA-R12-^125^I showed predominantly uptake in the liver, spleen, kidney, intestine, and tumor within the first 8 h p.i., but were rapidly cleared from all the tested organs at 24 h p.i., showing good biodegradability and rapid clearance. Mel@PSMA-R12-^125^I gradually accumulated in the tumor sites during the first 8 h p.i., showing good targeting properties to PCa tumor cells of the PSMA-targeted nanoparticles. These results showed that the self-assembled PSMA-targeted nanoparticles encapsulating melatonin, siAPE1, and/or ^125^I radioactive particles were efficient tumor-targeting therapies for PCa.

Although we found that Mel@PSMA-R12/siAPE1 does have good efficacy, its price is relatively expensive at present, and it is difficult to promote large-scale applications in the short term. In addition, it is still in the experimental animal stage and has not really been used in clinical research. It needs to be further broadened and developed by subsequent research.

## CONCLUSION

Our study showed that self-assembled nanoparticles PSMA-R12-^125^I had good PCa tumor cells-targeting properties and therapeutic functions with efficient co-delivery of siAPE1 and melatonin. The PSMA-targeted Mel@PSMA-R12-^125^I/siAPE1 nanocomposites had promising effectiveness in suppressing PCa tumor growth *in vivo*. Also, the self-assembled nanoparticles had good biodegradability and can provide a theoretical basis for patent applications.

## Figures and Tables

**Fig. (1):**
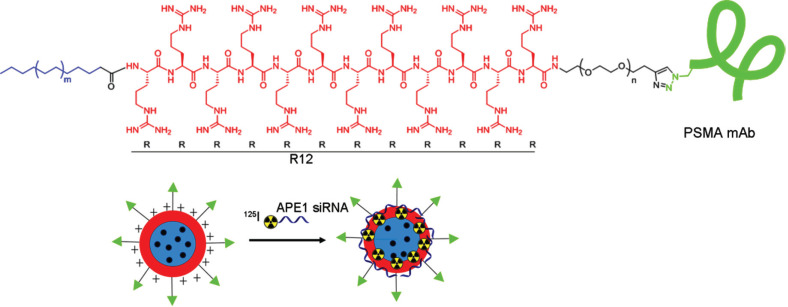
The artistic representation of the synthetic process for PSMA-R12 nanoparticles and Mel@PSMA-R12-^125^I/siAPE1 nanocomposites.

## Data Availability

The data and supportive information are available within the article.

## CURRENT AND FUTURE DEVELOPMENTS

PSMA-R12-^125^I encapsulating siAPE1 and melatonin is a promising alternative therapy for PCa.

## LIST OF ABBREVIATIONS

CT: Computed Tomography
PAGE: Polyacrylamide Gel Electrophoresis
PET: Positron Emission Tomography
PCa: Prostate Cancer
PSA: Prostate-specific Antigen
PSMA: Prostate-specific Membrane Antigen
